# Anti-Neoplastic Cytotoxicity of Gemcitabine-(C_4_-*amide*)-[anti-HER2/*neu*] in Combination with Griseofulvin against Chemotherapeutic-Resistant Mammary Adenocarcinoma (SKBr-3)

**DOI:** 10.4172/2161-0444.1000141

**Published:** 2013-06-29

**Authors:** CP Coyne, Toni Jones, Ryan Bear

**Affiliations:** Department of Basic Sciences, College of Veterinary Medicine at Wise Center, Mississippi State University, Mississippi State, Mississippi 39762, USA

**Keywords:** Anti-HER2/*neu*, Chemotherapeutic-resistant, Mammary adenocarcinoma, Covalent immunochemotherapeutic, Anti-neoplastic cytotoxicity, Tubulin/microtubule inhibitor, Dual combination anti-cancer therapy, Gemcitabine-(C_4_-*amide*)-[anti-HER2/*neu*], Griseofulvin

## Abstract

**Introduction:**

Gemcitabine is a pyrimidine nucleoside analog that becomes triphosphorylated and in this form it competitively inhibits cytidine incorporation into DNA strands. Diphosphorylated gemcitabine irreversibly inhibits ribonucleotide reductase thereby preventing deoxyribonucleotide synthesis. Functioning as a potent chemotherapeutic, gemcitabine decreases neoplastic cell proliferation and induces apoptosis which accounts for its effectiveness in the clinical treatment of several leukemia and carcinoma cell types. A brief plasma half-life due to rapid deamination, chemotherapeuticresistance and sequelae restricts gemcitabine utility in clinical oncology. Selective “targeted” gemcitabine delivery represents a molecular strategy for prolonging its plasma half-life and minimizing innocent tissue/organ exposure.

**Methods:**

A previously described organic chemistry scheme was applied to synthesize a UV-photoactivated gemcitabine intermediate for production of gemcitabine-(C_4_-*amide*)-[anti-HER2/*neu*]. Immunodetection analysis (Western-blot) was applied to detect the presence of any degradative fragmentation or polymerization. Detection of retained binding-avidity for gemcitabine-(C_4_-*amide*)-[anti-HER2/*neu*] was determined by cell-ELISA using populations of chemotherapeutic-resistant mammary adenocarcinoma (SKBr-3) that highly over-express the HER2/*neu* trophic membrane receptor. Anti-neoplastic cytotoxicity of gemcitabine-(C_4_-*amide*)-[anti-HER2/*neu*] and the tubulin/microtubule inhibitor, griseofulvin was established against chemotherapeutic-resistant mammary adenocarcinoma (SKBr-3). Related investigations evaluated the potential for gemcitabine-(C_4_-*amide*)-[anti-HER2/*neu*] in dual combination with griseofulvin to evoke increased levels of anti-neoplastic cytotoxicity compared to gemcitabine-(C_4_-*amide*)-[anti-HER2/*neu*].

**Results:**

Covalent gemcitabine-(C_4_-*amide*)-[anti-HER2/*neu*] immunochemotherapeutic and griseofulvin exerted anti-neoplastic cytotoxicity against chemotherapeutic-resistant mammary adenocarcinoma (SKBr-3). Covalent gemcitabine-(C_4_-*amide*)-[anti-HER2/*neu*] immunochemotherapeutic or gemcitabine in dual combination with griseofulvin created increased levels of anti-neoplastic cytotoxicity that were greater than was attainable with gemcitabine-(C_4_-*amide*)-[anti-HER2/*neu*] or gemcitabine alone.

**Conclusion:**

Gemcitabine-(C_4_-*amide*)-[anti-HER2/*neu*] in dual combination with griseofulvin can produce enhanced levels of anti-neoplastic cytotoxicity and potentially provide a basis for treatment regimens with a wider margin-of-safety. Such benefits would be possible through the collective properties of; [i] selective “*targeted*” gemcitabine delivery; [ii] relatively lower toxicity of griseofulvin compared to many if not most conventional chemotherapeutics; [iii] reduced total dosage requirements faciliated by additive or synergistic anti-cancer properties; and [iv] differences in sequelae for gemcitabine-(C_4_-*amide*)-[anti-HER2/*neu*] compared to griseofulvin functioning as a tubulin/microtubule inhibitor.

## Introduction

Monoclonal immunoglobulin preparations or pharmaceuticals with binding-avidity for HER2/*neu* (e.g. anti-HER2/*neu*: trastuzumab, pertuzumab) [1-5], EGFR (e.g. anti-EGFR: cetuximab, gefitinib) [6-9], immunoglobulin fractions with dual binding-avidity for both HER2/neu and EGFR (e.g. anti-HER2/*neu* and anti-EGFR properties: panitumumab) [8-11] or monoclonal immunoglobulin inhibitors of other trophic receptors can all be effective treatment options for cancer including forms of neoplasia affecting the breast, intestinal tract, lung and prostate. The obvious advantage of these preparations is their ability to function as an anti-cancer treatment modality that avoids many of the sequelae associated with conventional chemotherapeutics. Unfortunately, most monoclonal immunoglobulin-based therapies that inhibit the function of trophic membrane receptors are usually only capable of exerting cytostatic properties and are almost invariably plagued by an inability to evoke cytotoxic activity sufficient to effectively resolve most aggressive or advanced forms of neoplastic disease [12-17]. However, enhanced levels of anti-neoplastic cytotoxicity can be attained when monoclonal immunoglobulin-based biotherapies are applied in concert with conventional chemotherapeutics or other cancer treatement modalities [18,19].

The anthracyclines have traditionally been the class of chemotherapeutics most commonly bound covalently to (large) molecular platforms that can facilitate “selective” targeted delivery. Gemcitabine, in contrast to the anthracyclines, is a chemotherapeutic that has less frequently been covalently bound to large molecular weight platforms that can provide various biological properties [20,21] including selective “targeted” delivery [22,23]. Gemcitabine is a deoxycytidine nucleotide analog with a mechanism-of-action that is dependent upon intracellular triphosphoralation which allows it to substitute for cytidine during DNA transcription. In this capacity triphosphoralated gemcitabine both inhibits DNA polymerase biochemical activity and it becomes incorporated into DNA strands. A second mechanism-of-action involves gemcitabine inhibiting and inactivating ribonucleotide reductase in concert with suppression of deoxyribonucleotide synthesis, diminished DNA repair, and declines in DNA transcription. Each of these mechanisms-of-action contributes to initiating the onset of apoptosis. In clinical oncology, gemcitabine is administered for the treatment of certain leukemias and potentially different types of lymphoma in addition to a spectrum of adenocarcinomas and carcinomas affecting the lung (e.g. non-small cell), pancrease, bladder and esophogus. The plasma half-life for gemcitabine is brief because it is rapidly deaminated to an inactive metabolite that is then redily eliminated through renal excretion into the urine [24-26].

Despite general familiarity with the influence of anti-HER2/*neu* immunoglobulin on the viability and vitality of cancer cell populations and it’s application in clinical oncology, there is surprisingly little known about covalent gemcitabine-(anti-HER2/*neu*) immunochemotherapeutics and their potential to exert selectively “targeted” anti-neoplastic cytotoxicity against chemotherapeutic-resistant mammary adenocarcinoma [22,23]. Several distinct attributes can be realized through the molecular design and organic chemistry synthesis of a covalent gemcitabine immunochemotherapeutic that in part include the properties of selective “targeted” chemotherapeutic delivery, continual chemotherapeutic deposition, progressive intracellular chemotherapeutic accumulation, and extended plasma chemotherapeutic pharmacokinetic profiles. Presumably due steric hinderance phenomenon, gemcitabine covalently bound to large molecular weight platforms like immunoglobulin is also less vulnerable to MDR-1 (multi-drug resistance efflux pump) [27,28], or biochemical deamination by cytidine deaminase and deoxycytidylate deaminase (following gemcitabine phosphorylation). Covalently bonding gemcitabine to immunoglobulin or molecular ligands also provides opportunities for attaining additive or synergistic levels of anti-neoplastic cytotoxicity. One approach to attaining additive or synergistic anti-neoplastic cytotoxicity properties includes the utilization of large molecular weight platforms like anti-HER2/*neu*, anti-EGFR and similar monoclonal immunoglobulin fractions that provide a mechanism for simultaneously achieving selective “targeted” chemotherapeutic delivery and suppress biological vitality in neoplastic cell populations that are heavily dependent on trophic receptor over-expression.

Gemcitabine in clinical scenarios is frequently administered in combination with tubulin/microtubule inhibitor chemotherapeutics including the vinca alkaloids [29-32], taxanes [30,33,34], podophyllotoxins/etoposides [35-37], and monomethyl auristatin E (MMAE) [38]. Such combinations have commonly been administered for the therapeutic management of neoplastic conditions affecting the breast [29-33] pancrease [37] lung [36], in addition to lymphoproliferative disorders [38]. Clinical trials have been conducted to evaluate the efficacy of gemcitabine in combination with vinca alkaloid (2010: sarcomas) and Brentuximab Vedotin (2011: anaplastic large cell lymphoma, Hodgkin’s Lymphoma). The anti-fungal agent, griseofulvin has a mechanism-of-action highly analogous to the vinca alkaloids and other conventional tubulin/microtubule inhibitor chemotherapeutics but to date little is known about the anti-neoplastic cytotoxicity of this anti-fungal agent. In order to address this knowledge void and due to the potential to achieve elevated levels of efficacy and wider margins-of-safety, griseofulvin was evaluated for anti-neoplastic cytotoxicity against chemotherapeutic-resistant mammary carcinoma (SKBr-3) both alone and simultaneously in dual combination with gemcitabine-(C_4_-*amide*)-[anti-HER2/*neu*].

## Materials and Methods

### Synthesis of Gemcitabine-(C_4_-*amide*)-[anti-HER2/*neu*] Immunochemotherapeutic

#### Phase-I synthesis scheme for UV-photoactivated gemcitabine-(C_4_-amide) intermediates

**Figure 1:**
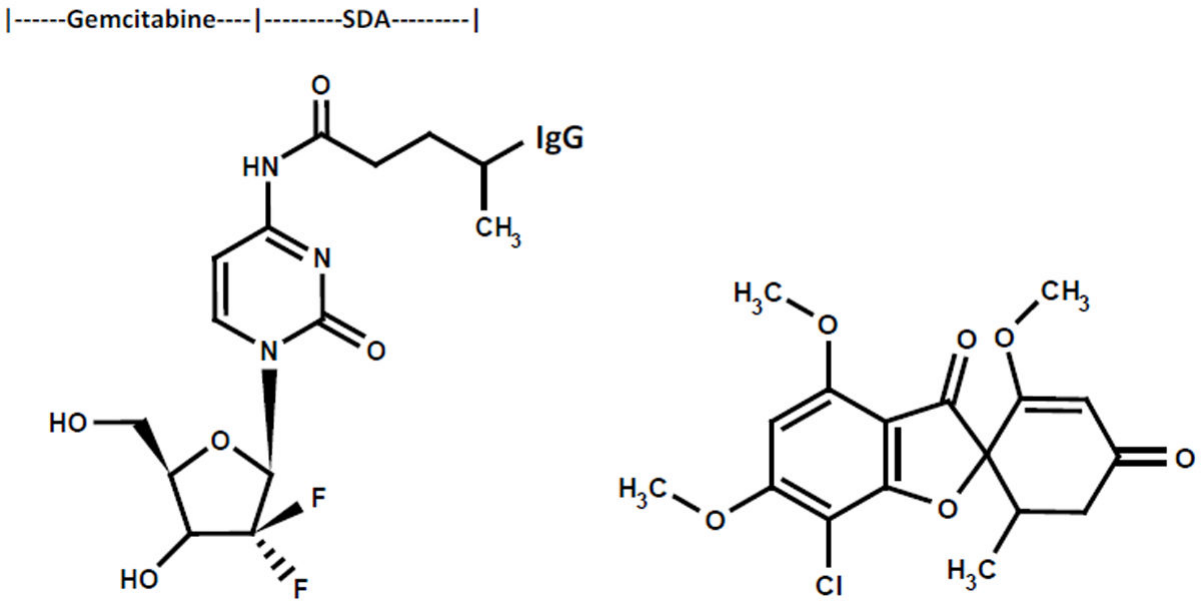
Molecular design and chemical structures. *Legend* (*Left Panel*) covalent immunochemotherapeutic, gemcitabine-(C_4_-*amide*)-[anti-HER2/*neu*] synthesized utilizing a 2-stage organic chemistry reaction scheme that initially generates a gemcitabine UV-photoactivated intermediate; (*Right Panel*) griseofulvin capable of functioning as an alternative tubulin/microtubule inhibitor chemotherapeutic agent.

The cytosine-like C_4_-*amine* of gemcitabine (0.738 mg, 2.80×10^-3^ mmoles) was reacted at a 2.5:1 molar-ratio with the amine-reactive *N*-hydroxysuccinimide ester “leaving” complex of succinimidyl 4,4-azipentanoate (0.252 mg, 1.12×10^-3^ mmoles) in the presence of triethylamine (TEA 50 mM final concentration) utilizing dimethylsulfoxide as an anhydrous organic solvent system (Figure 1). The reaction mixture formulated from stock solutions of gemcitabine and succinimidyl 4,4-azipentanoate was continually stirred gently at 25°C over a 4-hour incubation period in the dark and protected from exposure to light. The relatively long incubation period of 4 hours was utilized to maximize ester group degradation associated with any residual succinimidyl 4,4-azipentanoate that may not of reacted in the first 30 to 60 minutes with the C_4_ cytosine-like mono-amine group of gemcitabine.

#### Phase-II synthesis scheme for covalent gemcitabine-(C_4_-amide)-[anti-HER2/neu] immunochemotherapeutic utilizing a UV-photoactivated gemcitabine intermediate

Immunoglobulin fractions of anti-HER2/*neu* (1.5 mg, 1.0×10^-5^ mmoles) in buffer (PBS: phosphate 0.1, NaCl 0.15 M, EDTA 10 mM, pH 7.3) were combined at a 1:10 molar-ratio with the UV-photoactivated gemcitabine-(C_4_-*amide*) intermediate (*Phase-1 end product*) and allowed to gently mix by constant stirring for 5 minutes at 25°C in the dark. The photoactivated group of the gemcitabine-(C_4_-*amide*) intermediate was reacted with side chains of amino acid residues within the sequence of anti-HER2/*neu* monoclonal immunoglobulin during a 15 minute exposure to UV light at 354 nm (reagent activation range 320-370 nm) in combination with constant gentle stirring (Figure 1). Residual gemcitabine was removed from gemcitabine-(C_4_-*amide*)-[anti-HER2/*neu*] applying micro-scale column chromatography following pre-equilibration of exchange media with PBS (phosphate 0.1, NaCl 0.15 M, pH 7.3). Residual gemcitabine was removed from gemcitabine-(C_4_-*amide*)-[anti-HER2/*neu*] applying micro-scale column chromatography following pre-equilibration of exchange media with PBS (phosphate 0.1, NaCl 0.15 M, pH 7.3).

### Molecular analysis and characterization

#### General analysis

Approximation of the amount of non-covalently bound gemcitabine contained within the covalent gemcitabine-(C_4_-*amide*)-[anti-HER2/*neu*] immunochemotherapeutic following separation by column chromatography was determined by measuring absorbance at 265-267 nm [39,40] of the resulting supernatant after precipitation of gemcitabine-immunochemotherapeutics with either chloroform [41-43] or methanol:acetonitrile (1:9 v/v) with measurements compared to original known concentrations [23]. Determination of the immunoglobulin concentration for the covalent gemcitabine-(C_4_-*amide*)-[anti-HER2/*neu*] immunoconjugates was determined by measuring absorbance at 280 nm in combinations with utilizing a 235 nm -vs- 280 nm standardized reference curve in order to accommodate for any potential absorption profile over-lap at 280 nm between gemcitabine and immunoglobulin [22,23,44-47].

#### Molecular mass/Size-dependent separation by non-reducing SDS-PAGE

The covalent gemcitabine-(C_4_-*amide*)-[anti-HER2/*neu*] immunochemotherapeutic and anti-HER2/*neu* immunoglobulin fraction reference control were adjusted to a standardized protein concentration of 60 μg/ml and then combined 50/50 v/v with conventional SDS-PAGE sample preparation buffer (Tris/glycerol/bromphenyl blue/SDS) formulated without 2-mercaptoethanol or boiling. Each covalent immunochemotherapeutic, the reference control immunoglobulin fraction (0.9 μg/well) and a mixture of pre-stained reference control molecular weight markers were then developed by non-reducing SDS-PAGE (11% acrylamide) performed at 100 V constant voltage at 3°C for 2.5 hours.

#### Western-blot immunodetection analyses

Covalent gemcitabine-(C_4_-*amide*)-[anti-HER2/*neu*] immunochemotherapeutic following mass/size-dependent separation by non-reducing SDS-PAGE were equilibrated in tank buffer devoid of methanol. Mass/size-separated gemcitabine and anthracycline anti-HER2/*neu* immunochemotherapeutics contained in acrylamide SDS-PAGE gels were then transferred laterally onto sheets of nitrocellulose membrane at 20 volts (constant voltage) for 16 hours at 2° to 3°C with the transfer manifold packed in crushed ice.

Nitrocellulose membranes with laterally-transferred immunochemotherapeutics were then equilibrated in Tris buffered saline (TBS: Tris HCl 0.1 M, NaCl 150 mM, pH 7.5, 40 ml) at 4°C for 15 minutes followed by incubation in TBS blocking buffer solution (Tris 0.1 M, pH 7.4, 40 ml) containing bovine serum albumin (5%) for 16 hours at 2° to 3°C applied in combination with gentle horizontal agitation. Prior to further processing, nitrocellulose membranes were vigorously rinsed in Tris buffered saline (Tris 0.1 M, pH 7.4, 40 ml, n=3x).

Rinsed BSA-blocked nitrocellulose membranes developed for Western-blot (immunodetection) analyses were incubated with biotinylated goat anti-murine IgG (1:10,000 dilution) at 4°C for 18 hours applied in combination with gentle horizontal agitation. Nitrocellulose membranes were then vigorously rinsed in TBS (pH 7.4, 4°C, 50 ml, n=3) followed by incubation in blocking buffer (Tris 0.1 M, pH 7.4, with BSA 5%, 40 ml). Blocking buffer was decanted from nitrocellulose membrane blots which were then rinsed in TBS (pH 7.4, 4°C, 50 ml, n=3) before incubation with strepavidin-HRPO (1:100,000 dilution) at 4°C for 2 hours applied in combination with gentle horizontal agitation. Prior to chemiluminescent development nitrocellulose membranes were vigorously rinsed in Tris buffered saline (Tris 0.1 M, pH 7.4, 40 ml, n=3). Development of nitrocellulose membranes by chemiluminescent autoradiography following processing with conjugated HRPO-strepavidin required incubation in HRPO chemiluminescent substrate (25°C; 5 to 10 mins.). Autoradiographic images were acquired by exposing radiographic film (Kodak BioMax XAR) to nitrocellulose membranes sealed in transparent ultraclear re-sealable plastic bags.

### Chemotherapeutic-resistant mammary adenocarcinoma

#### Mammary adenocarcinoma tissue culture

Chemotherapeutic-resistant human mammary adenocarcinoma (SKBr-3) was utilized as an *ex-vivo* neoplasia model. Mammary adenocarcinoma (SKBr-3) characteristically over-expresses epidermal growth factor receptor 1 (EGFR, ErbB-1, HER1) and highly over-expresses epidermal growth factor receptor 2 (EGFR2, HER2/*neu*, ErbB-2, CD340, p185) at 2.2×10^5^/cell and 1×10^6^/cell respectively.

Populations of the mammary adenocarcinoma (SKBr-3) cell line were propagated in 150-cc^2^ tissue culture flasks containing McCoy’s 5a Modified Medium supplemented with fetal bovine serum (10% v/v) and penicillin-streptomycin at a temperature of 37°C under a gas atmosphere of air (95%) and carbon dioxide (5% CO_2_). Tissue culture media was not supplemented with growth factors, growth hormones or other growth stimulants of any type. Investigations were performed using mammary adenocarcinoma (SKBr-3) monolayer populations at a >85% level of confluent.

#### Cell-ELISA total membrane-bound immunoglobulin assay

Cell suspensions of mammary adenocarcinoma (SKBr-3) were seeded into 96-well microtiter plates in aliquots of 2×10^5^ cells/well and allowed to form a confluence adherent monolayer over a period of 48 hours. Growth media within each individual wells was then removed manually by pipette and the monolayers were serially rinsed (n=3) with PBS followed by stabilization of adherent (SKBr-3) cellular monolayers onto the plastic surface of 96-well plates with paraformaldehyde (4% in PBS, 15 minutes). Stabilized (SKBr-3) monolayers were then incubated with gemcitabine-(C_4_-*amide*)-[anti-HER2/*neu*] immunoconjugate formulated at gradient concentrations of 0.1, 0.25, 0.5, 1.0, 5.0 and 10 μg IgG/ml in tissue culture growth media (200 μl/well). Mammary adenocarcinoma (SKBr-3) cellular monolayer were then subjected to direct contact with gemcitabine-(C_4_-*amide*)-[anti-HER2/*neu*] at 37°C over an incubation period of 3-hours using a gas atmosphere of air (95%) and carbon dioxide (5% CO_2_). Development of stabilized mammary adenocarcinoma (SKBr-3) monolayers following serial rinsing with PBS (n=3) entailed incubation with β-galactosidase conjugated goat anti-mouse IgG (1:500 dilution) for 2 hours at 25°C with residual unbound immunoglobulin removed by serial rinsing with PBS (n=3). Final cell ELISA development required serial rinsing (n=3) of stabilized (SKBr-3) monolayers with PBS followed by incubation with nitrophenyl-β-D-galactopyranoside substrate (100 μl/well of ONPG formulated fresh at 0.9 mg/ml in PBS pH 7.2 containing MgCl_2_ 10 mM, and 2-mercaptoethanol 0.1 M). Absorbance within each individual well was measured at 410 nm (630 nm reference wavelength) after incubation at 37°C for a period of 15 minutes.

#### Cell vitality stain-based assay: anti-neoplastic cytotoxicity

Covalent gemcitabine-(C_4_-*amide*)-[anti-HER2/*neu*] immunochemotherapeutic was formulated in growth media at final standardized chemotherapeutic-equivalent concentrations of 10^-10^, 10^-9^, 10^-8^, 10^-7^, and 10^-6^ M. Similarly, griseofulvin was formulated in growth media at 1.0, 10, 20, 40, 60, 80 and 100 μM (final concentrations). The covalent immunochemotherapeutic or griseofulvin was then transferred in triplicate into 96-well microtiter plates containing mammary adenocarcinoma (SKBr-3) monolayers (growth media 200 μl/well) and allowed to incubate in direct contact with cell populations for a period of either 96 or 182-hours (37°C under a gas atmosphere of 95% air and 5% carbon dioxide/ CO_2_). Each individual 96-well compartment contained at least a minimum of 90% (v/v) growth media. Incubation periods of greater than 96-hours required replenishing mammary adenocarcinoma (SKBr-3) populations with fresh tissue culture media formulated with or without covalent gemcitabine-immunochemotherapeutics or griseofulvin tubulin/microtubule inhibitor as indicated.

Cytotoxic potency of gemcitabine-(C_4_-*amide*)-[anti-HER2/*neu*] or griseofulvin was measured by removing all contents within the 96-well microtiter plates manually by pipette followed by serial rinsing of monolayers with PBS (n=3) and incubation with 3-[4,5-dimethylthiazol-2-yl]-2,5-diphenyl tetrazolium bromide vitality stain reagent formulated in RPMI-1640 growth media devoid of pH indicator or bovine fetal calf serum (MTT: 5 mg/ml). During a 3-to-4 hour incubation period under a gas atmosphere of air (95%) and carbon dioxide (5% CO_2_) the enzyme mitochondrial succinate dehydrogenase was allowed to convert the MTT vitality stain reagent to navy-blue formazone crystals within the cytosol of mammary adenocarcinoma (SKBr-3) populations (some reports suggest that NADH/NADPH-dependent cellular oxidoreductase enzymes may also be involved in the conversion process). Contents of the 96-well microtiter plate were then removed followed by serial rinsing with PBS (n=3). The resulting blue intracellular formazone crystals were dissolved with DMSO (300 μl/well) and then the spectrophotometric absorbance of the blue-colored supernantant measured at 570 nm using a computer integrated microtiter plate reader.

## Results

### Molar-incorporation-index

Size-separation of gemcitabine-(C_4_-*amide*)-[anti-HER2/*neu*] by buffer exchange column chromatography consistently yielded covalent immunochemotherapeutic preparations that contain <4.0% of residual non-covalently bound chemotherapeutic [22,23,44-47]. Small residual amounts of non-covalently bound chemotherapeutic that remain within covalent immunochemotherapeutic preparations is generally accepted to not be available for further removal through any additional sequential column chromatography separations [48]. Calculations estimated a 2.78 molar-incorporation index for covalent gemcitabine-(C_4_-*amide*)-[anti-HER2/*neu*] immunochemotherapeutic.

### Molecular weight profile analysis

**Figure 2:**
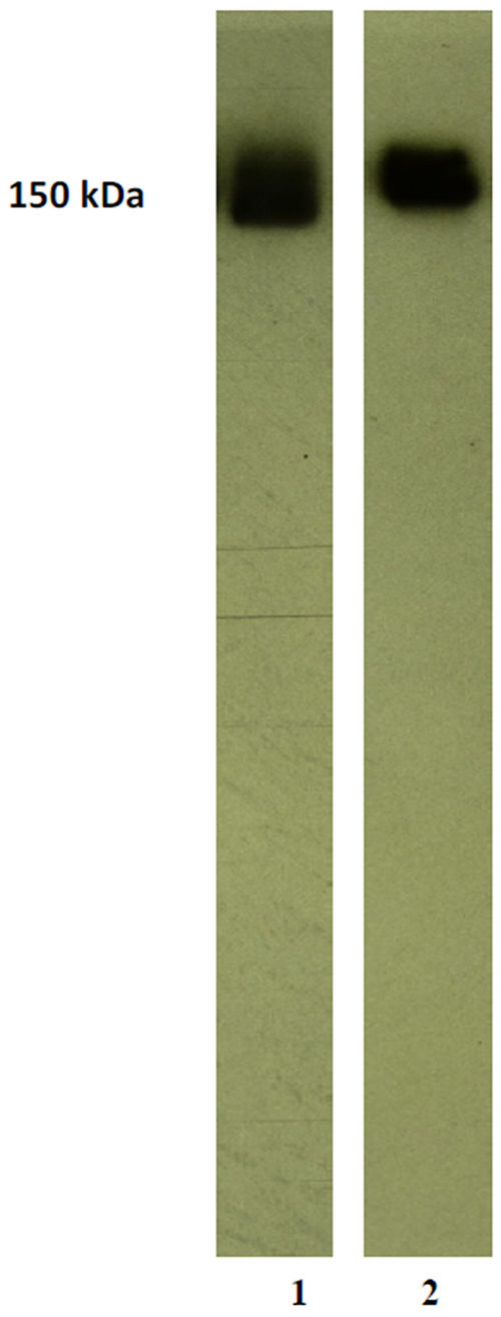
Characterization of the major molecular weight profile for covalent gemcitabine-(C_4_-*amide*)-[anti-HER2/*neu*] immunochemotherapeutics compared to anti-HER2/*neu* monoclonal immunoglobulin. *Legends*: (*Lane-1)* murine anti-human HER2/*neu* monoclonal immunoglobulin reference control; and *(Lane-2)* covalent gemcitabine-(C_4_-*amide*)-[anti-HER2/*neu*] immunochemotherapeutic. Covalent gemcitabine immunochemotherapeutic and anti-HER2/neu monoclonal immunoglobulin were size-separated by non-reducing SDS-PAGE followed by lateral transfer onto sheets of nitrocellulose membrane to facilitate detection with biotinylated goat anti-mouse IgG immunoglobulin. Subsequent analysis entailed incubation of nitrocellulose membranes with strepavidin-HRPO in combination with the use of a HRPO chemiluminescent substrate for the acquisition of autoradiography images.

Mass/size separation of covalent gemcitabine-(C_4_-*amide*)-[anti-HER2/*neu*] immunochemotherapeutic by SDS-PAGE in combination with immunodetection analysis (Western blot) and chemiluminescent autoradiography recognized a single primary condensed band of 150-kDa between a molecular weight range of 5.0-kDa to 450-kDa (Figure 2). Patterns of low-molecular-weight fragmentation from hydrolytic or enzymatic degradation, or evidence of large-molecular weight polymerization of immunoglobulin fractions were not detected (Figure 2). The observed molecular weight of 150-kDa for gemcitabine-(C_4_-*amide*)-[anti-HER2/*neu*] directly corresponds with the known molecular weight/mass of reference control anti-HER2/*neu* monoclonal immunoglobulin fractions (Figure 2). Analogous results have been reported for similar covalent immunochemotherapeutics. [22,23,44-47,49,50]

### Cell-binding analysis

**Figure 3:**
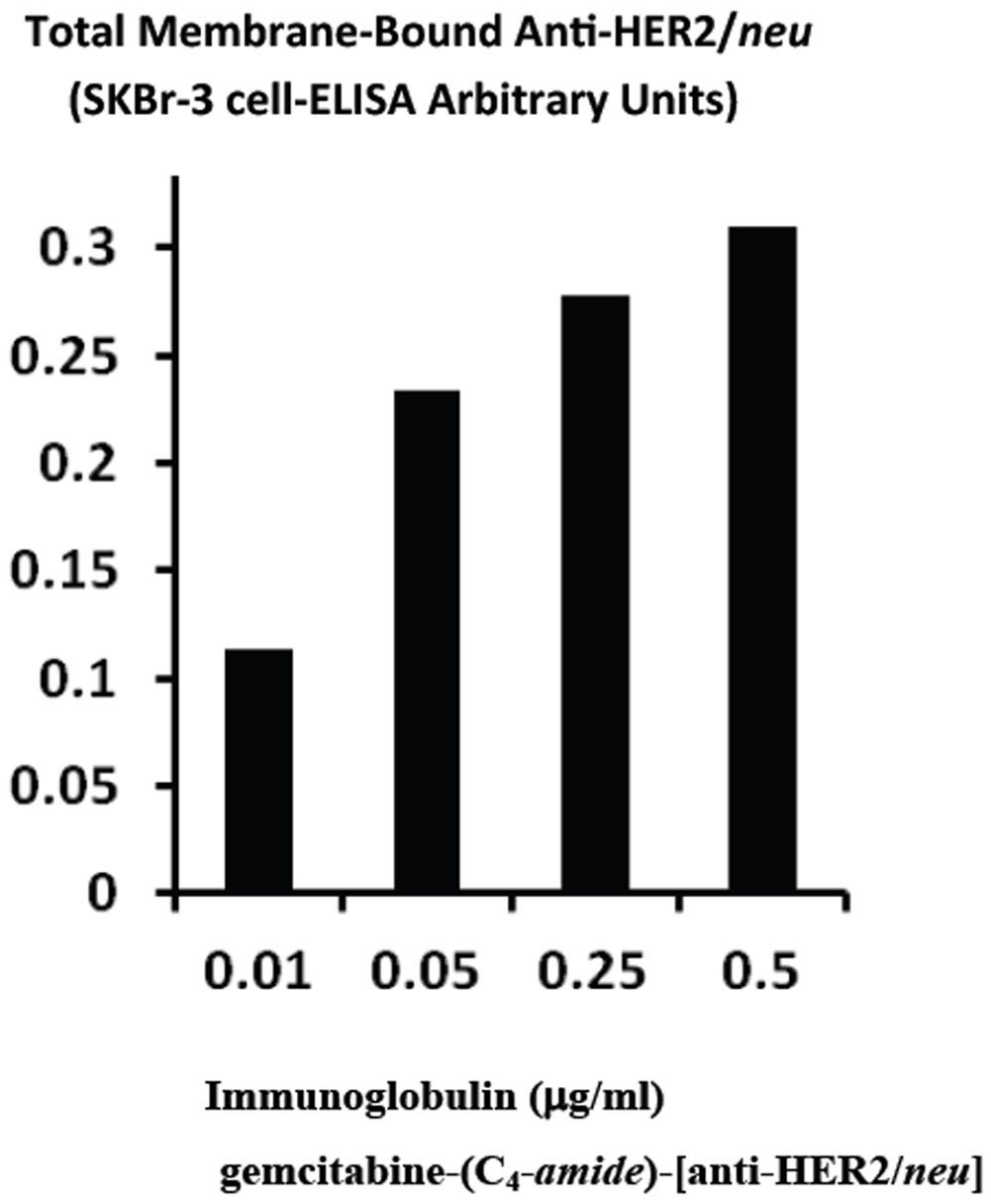
Detection of total anti-HER2/*neu* immunoglobulin in the form of gemcitabine-(C_4_-*amide*)-[anti-HER2/*neu*] bound to HER2/*neu* on the exterior surface membrane of chemotherapeutic-resistant mammary adenocarcinoma (SKBr-3). Covalent gemcitabine-(C_4_-*amide*)-[anti-HER2/*neu*] immunochemotherapeutic was incubated with monolayer populations of mammary adenocarcinoma (SKBr-3) over a 4-hour period and cell-ELISA was applied to measure total exterior surface membrane bound immunoglobulin.

Total bound immunoglobulin in the form of gemcitabine-(C_4_-*amide*)-[anti-HER2/*neu*] on the external surface membrane of adherent mammary adenocarcinoma (SKBr-3) populations was measured by cell-ELISA (Figure 3). Greater total membrane-bound gemcitabine-(C_4_-*amide*)-[anti-HER2/*neu*] was detected with progressive increases in standardized total immunoglobulin-equivalent concentrations formulated at 0.010, 0.025, 0.050, 0.250, and 0.500 μg IgG/ml (Figure 3). The cell-ELISA profiles therefore serve to validate the retained selective binding-avidity of gemcitabine-(C_4_-*amide*)-[anti-HER2/*neu*] for HER2/*neu* receptor sites highly over-expressed at 1×10^6^/cell on the exterior surface membrane of mammary adenocarcinoma (SKBr-3) populations (Figure 3) [22].

#### Anti-neoplastic cytotoxicity analysis

**Figure 4:**
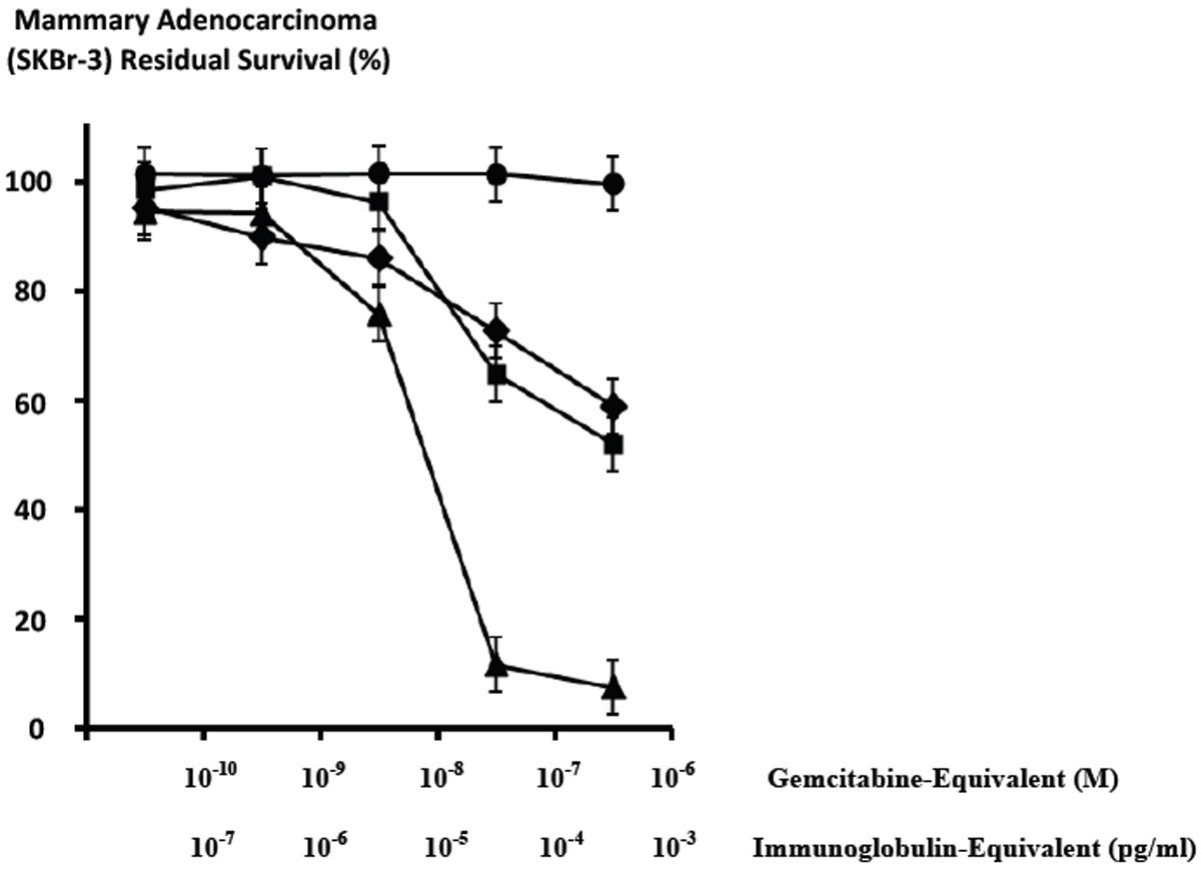
Differences in anti-neoplastic cytotoxicity for gemcitabine-(C_4_-*amide*)-[anti-HER2/*neu*], gemcitabine alone, anti-HER2/*neu* monoclonal immunoglobulin as a function of time. *Legends*: (◆) covalent gemcitabine-(C_4_-*amide*)-[anti-HER2/*neu*] immunochemotherapeutic following a 182-hours incubation period; (■) gemcitabine chemotherapeutic following a 96-hour incubation period; (□) gemcitabine chemotherapeutic following a 182-hour incubation period; and (●) anti-HER2/*neu* monoclonal immunoglobulin Chemotherapeutic-resistant mammary adenocarcinoma (SKBr-3) monolayer populations were incubated with covalent gemcitabine-(C_4_-*amide*)-[anti-HER2/*neu*] and gemcitabine formulated in triplicate at gradient gemcitabine-equivalent concentrations. Anti-neoplastic cytotoxicity was measured using a MTT cell vitality assay relative to matched negative reference controls.

Gemcitabine-(C_4_-*amide*)-[anti-HER2/*neu*] exerted a 41.1% maximum level of selective “targeted” anti-neoplastic cytotoxicity (58.9% residual survival) against chemotherapeutic-resistant mammary adenocarcinoma (SKBr-3) at a gemcitabine-equivalent concentration of 10^-6^ M with progressive increases from 14% to 41.1% (86.0% and 58.9% residual survival) detected between 10^-8^ M and 10^-6^ M respectively (Figure 4).

Anti-neoplastic cytotoxicity profiles for gemcitabine-(C_4_-*amide*)-[anti-HER2/*neu*] after a 182-hour incubation period were highly analogous to gemcitabine chemotherapeutic following a 72-hour incubation period at the gemcitabine-equivalent concentrations of 10^-10^, 10^-9^, 10^-8^, 10^-7^ and 10^-6^ M (Figure 4). Gemcitabine at 182-hours produced rapid increases in anti-neoplastic cytotoxicity from 5.8% to 88.3% (94.2% and 11.7% residual survival) at and between the gemcitabine equivalent concentrations of 10^-9^ M and 10^-7^ M respectively (Figure 4) Maximum anti-neoplastic cytotoxicity for gemcitabine following an 182-hour incubation period at the gemcitabine-equivalent concentrations of 10^-6^ M was 92.5% (7.5% residual survival) respectively (Figure 4). Anti-neoplastic cytotoxicity for gemcitabine-(C_4_-*amide*)-[anti-HER2/*neu*] was detectably lower based on observed values of 27.3%% and 40.1% (72.7% and 58.9% residual survival) at 10^-7^ M and 10^-6^ M respectively (Figure 4) [22]. Monoclonal anti-HER2/*neu* immunoglobulin fractions alone did not exert detectable levels of *ex-vivo* anti-neoplastic cytotoxicity against chemotherapeutic-resistant mammary adenocarcinoma (SKBr-3) which is in direct accord with descriptions from previous investigations for anti-HER2/neu [22,23,44-47,50-54] and anti-EGFR [44] at 0-to-182 hours in populations of several different neoplastic cell types (Figure 4).

**Figure 5:**
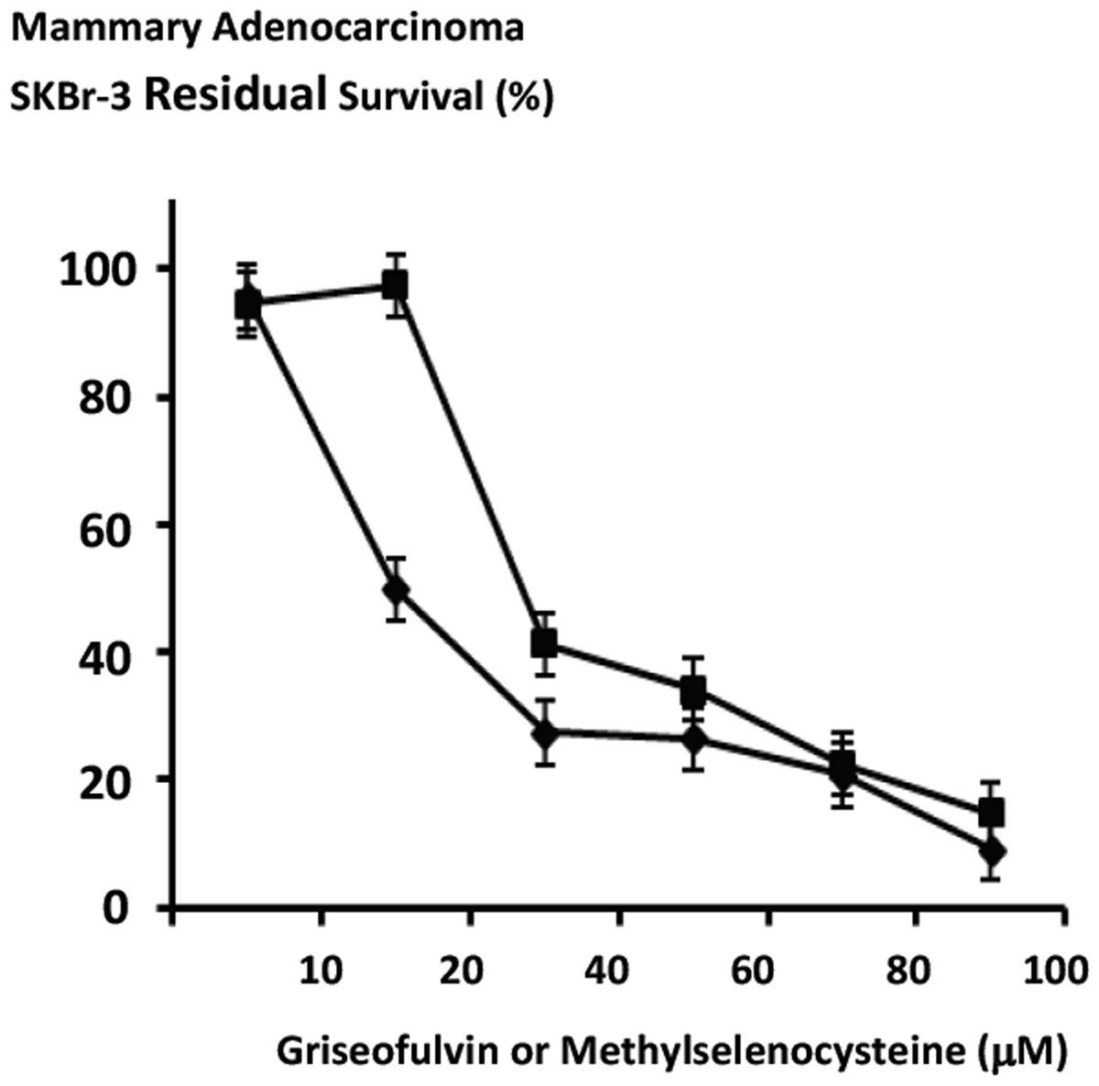
Relative anti-neoplastic cytotoxicity of the tubulin/microtubule inhibitor, griseofulvin compared to methylselencysteine against chemotherapeutic-resistant mammary adenocarcinoma. *Legend*: (◆) griseofulvin; and (■) methyselenocysteine. Mammary adenocarcinoma (SKBr-3) monolayer populations were incubated 96-hours with griseofulvin or methylselenocysteine formulated at gradient concentrations and anti-neoplastic cytotoxicity measured as a function of MTT cell vitality stain intensity relative to matched negative reference controls.

The anti-fungal tubulin/microtubule inhibitor, griseofulvin and methylselenocysteine both exerted detectable levels of anti-neoplastic cytotoxicity in a concentration-dependent manner against chemotherapeutic-resistant mammary adenocarcinoma (SKBr-3) when formulated at final concentrations that ranged between 0 μM to 100 μM (Figure 5). Methylselenocysteine created rapid, progressive increases in mean anti-neoplastic cytotoxicity from 2.6% to 58.7% (97.4% to 41.3% residual survival) at and between the concentrations of 20 μM and 40 μM with peak levels of 85.2% (14.8% residual survival) detected at a final concentration of 100 μM after a 96-hour incubation challenge (Figure 5). Griseofulvin created rapid, progressive elevations in mean anti-neoplastic cytotoxicity from 4.3% to 72.6% (95.7% to 27.4% residual survival) at and between the final concentrations of 0 μM and 40 μM with peak levels of 90.8% (9.2% residual survival) observed at the final concentration of 100 μM after a 96-hour incubation challenge (Figure 5).

**Figure 6:**
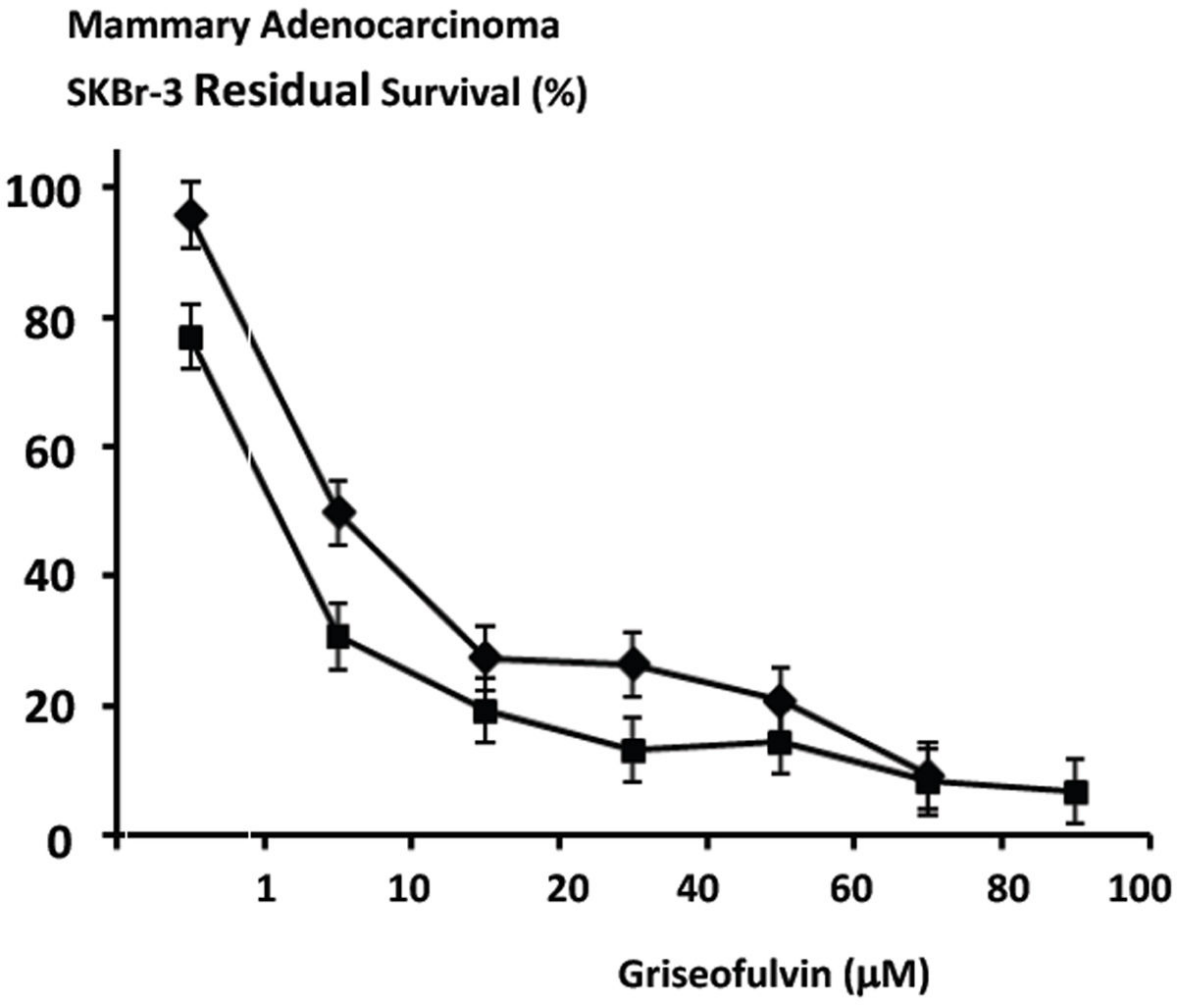
Relative anti-neoplastic cytotoxicity of griseofulvin functioning as a tubulin/microtubule inhibitor against chemotherapeutic-resistant mammary adenocarcinoma as a function of challenge duration. *Legend*: (■) griseofulvin following an incubation period of 182-hours, and (◆) griseofulvin following an incubation period of 96-hours. Mammary adenocarcinoma SKBr-3 monolayer populations were incubated with covalent gemcitabine immunochemotherapeutics. Cytotoxicity was measured applying the MTT cell vitality assay relative to matched negative reference controls.

Griseofulvin at the 96-hour and 182-hour incubation periods (direct contact) produced relatively rapid increases in anti-neoplastic cytotoxicity from 4.3% and 3.2% (95.6% and 96.8% residual survival) to 72.6% and 86.8% (27.4% and 13.2% residual survival) at and between the griseofulvin-equivalent concentrations of 0 μM and 40 μM respectively. Compared to the 96-hour incubation period, a greater level of anti-neoplastic cytotoxicity was detected with a longer 182-hour griseofulvin incubation period particularly at the griseofulvin-equivalent concentrations of 20 μM, 40 μM, 60 μM and 80 μM (Figure 6). The largest differences detected for anti-neoplastic cytotoxicity between the 96-hour compared to the 182-hour incubation periods (direct contact) were 50.2% versus 80.8% (49.8% and 19.2% residual survival) measured at the griseofulvin-equivalent concentration of 20 mM respectively. Very gradual increases and near maximum levels of anti-neoplastic cytotoxicity for griseofulvin were found at the griseofulvin-equivalent concentrations of 40 μM, 60 μM, 80 μM, and 100 μM (Figure 6). Maximum anti-neoplastic cytotoxicity for gemcitabine at the 96-hour and 182-hour incubation periods were 90.8% versus 93.2% (9.2% and 6.8% residual survival) at the griseofulvin-equialent concentration of 100 μM respectively. Due to the rapid increase in anti-neoplastic cytotoxicity for griseofulfin at and between 1 μM and 20 μM concentrations of 1 μM and 10 μM were subsequently evaluated (Figure 6).

**Figure 7:**
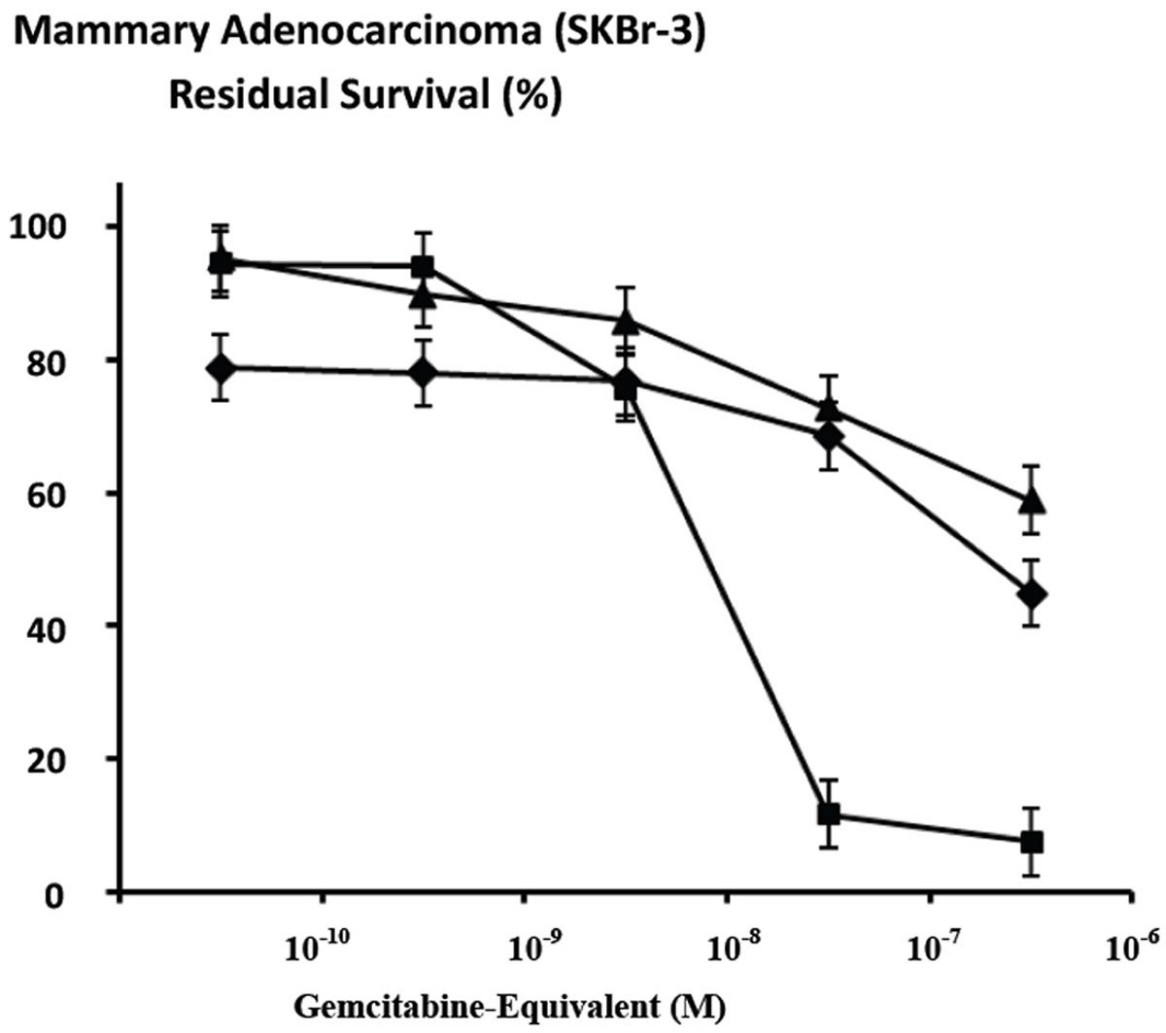
Relative enhancement of the anti-neoplastic cytotoxicity of gemcitabine-(C_4_-*amide*)-[anti-HER2/*neu*] in dual combination with griseofulvin against chemotherapeutic-resistant mammary adenocarcinoma. *Legend*: (◆) gemcitabine-(C_4_-*amide*)-[anti-HER2/*neu*] with griseofulvin; (□) gemcitabine-(C_4_-*amide*)-[anti-HER2/*neu*]; and (■) gemcitabine alone. Chemotherapeutic-resistant mammary adenocarcinoma (SKBr-3) monolayer populations were incubated with covalent gemcitabine-(C_4_-*amide*)-[anti-HER2/*neu*] immunochemotherapeutic (+/-grisofulvin 15 μM fixed-concentration) formulated in triplicate at gradient concentrations. Anti-neoplastic cytotoxicity was measured using a MTT cell vitality assay relative to matched negative reference controls.

Gemcitabine-(C_4_-*amide*)-[anti-HER2/*neu*] in combination with griseofulvin (15 μM fixed-concentration) compared to just gemcitabine-(C_4_-*amide*)-[anti-HER2/*neu*] alone resulted in anti-neoplastic cytotoxicity profiles there were similar but there was an obvious trend for the dual combination to exert greater activity (Figure 7). Anti-neoplastic cytotoxicity of gemcitabine-(C_4_-*amide*)-[anti-HER2/*neu*] in dual combination with griseofulvin (15 μM fixed-concentration) progressively increased from 24.3% to a maximum of 55.1% (75.8% and 44.9% residual survival) at and between the gemcitabine-equivalent concentrations of 10^-8^ M and 10^-6^ M respectively (Figure 7). In contrast, gemcitabine-(C_4_-*amide*)-[anti-HER2/*neu*] alone displayed progressive increases in anti-neoplastic cytotoxicity from 4.7% to a maximum of 41.1% (95.3% and 58.9% residual survival) at and between the gemcitabine-equivalent concentrations of 10^-10^ M and 10^-6^ M respectively (Figure 7).

The relative anti-neoplastic cytotoxicity of gemcitabine-(C_4_-*amide*)-[anti-HER2/*neu*] in dual combination with griseofulvin (15 μM fixed-concentration) was compared to that of gemcitabine alone at and between the gemcitabine-equivalent concentrations 10^-10^ M and 10^-6^ M (Figure 7). Anti-neoplastic cytotoxicity of gemcitabine-(C_4_-*amide*)-[anti-HER2/*neu*] in dual combination with griseofulvin (15 μM fixed-concentration) progressively increased from 24.3% to a maximum of 55.1% (75.8% and 44.9% residual survival) at and between the gemcitabine-equivalent concentrations of 10^-8^ M and 10^-6^ M respectively (Figure 7). Gemcitabine alone at 182-hours by comparison produced rapid increases in anti-neoplastic cytotoxicity from 5.8% to 88.3% (94.2% and 11.7% residual survival) at and between the gemcitabine equivalent concentrations of 10^-9^ M and 10^-7^ M respectively (Figure 7) Maximum anti-neoplastic cytotoxicity for gemcitabine was 92.5% (7.5% residual survival) at the gemcitabineequivalent concentration of 10^-6^ M (Figure 7). Both gemcitabine-(C_4_-*amide*)-[anti-HER2/*neu*] in dual combination with griseofulvin (15 μM fixed-concentration), and gemcitabine alone produced essentially identical levels of anti-neoplastic cytotoxicity at the gemcitabine-equivalent concentration of 10^-8^ M, but very similar levels at 10^-10^ M and 10^-9^ M (Figure 7). The dual combination of gemcitabine-(C_4_-*amide*)-[anti-HER2/*neu*] with griseofulvin (15 μM fixed-concentration) produced a detectably greater level of anti-neoplastic cytotoxicity than gemcitabine alone at the gemcitabine-equivalent concentrations 10^-10^ M and 10^-9^ M (Figure 7). Anti-neoplastic cytotoxicity at higher chemotherapeutic concentrations revealed that gemcitabine alone was detectably more potent than gemcitabine-(C_4_-*amide*)-[anti-HER2/*neu*] in dual combination with griseofulvin (15 μM fixed-concentration) at the gemcitabine-equivalent concentrations of 10^-7^ M (88.3% -versus- 31.4%) and 10^-6^ M (92.5% -versus- 55.1%) respectively (Figure 7).

**Figure 8:**
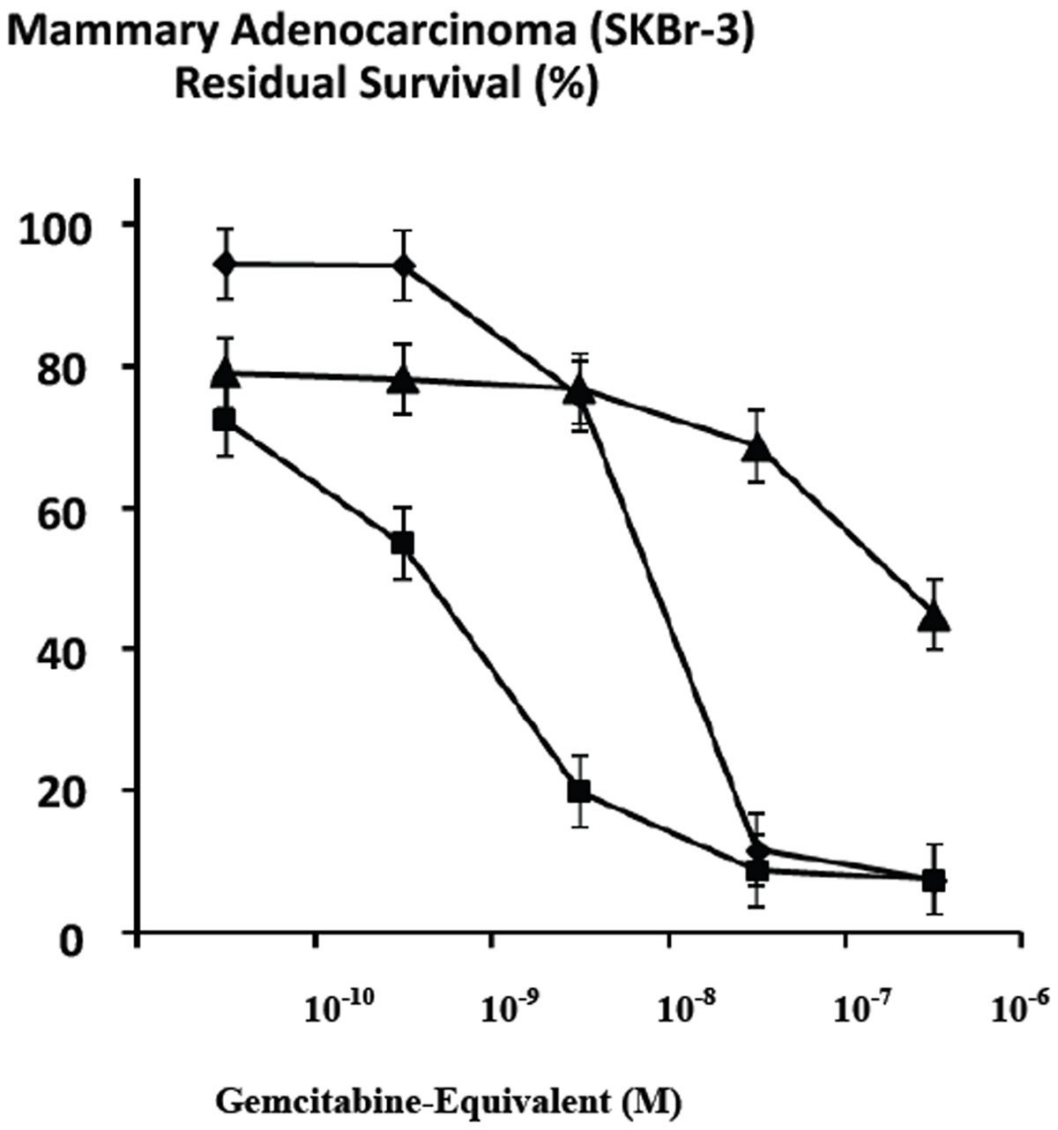
Relative anti-neoplastic cytotoxicity of gemcitabine in dual combination griseofulvin compared to gemcitabine alone against chemotherapeutic-resistant mammary adenocarcinoma. *Legend*: (■) gemcitabine with griseofulvin; (◆) gemcitabine alone; and (□) gemcitabine-(C_4_-*amide*)-[anti-HER2/*neu*] with griseofulvin. Chemotherapeutic-resistant mammary adenocarcinoma (SKBr-3) monolayer populations were incubated for 182-hours with gemcitabine (+/-grisofulvin 15 μM fixed-concentration) formulated in triplicate at gradient concentrations. Anti-neoplastic cytotoxicity was measured using a MTT cell vitality assay relative to matched negative reference controls.

Gemcitabine in dual combination with griseofulvin (15 μM fixed-concentration) was compared to gemcitabine alone in order to determine their relative anti-neoplastic cytotoxicity against chemotherapeutic resistant mammary adenocarcinoma (SKBr-3) populations (Figure 8). Greater anti-neoplastic cytotoxicity for gemcitabine in dual combination with griseofulin (15 μM fixed-concentration) compared to just gemcitabine alone was detected at the gemcitabine-equivalent concentrations of 10^-10^ M (27.8% -versus- 5.6%), 10^-9^ M (45.1% -versus- 3.8%), and 10^-8^ M (80.1% -versus- 24.3%) respectively (Figure 8). Essentially identical and near-maximum or maximum levels of anti-neoplastic cytotoxicity were detected for gemcitabine in dual combination with griseofulvin (15 μM fixed-concentration) compared to gemcitabine alone at the gemcitabine-equivalent concentrations of 10^-7^ M (91.3% and 88.3%) and 10^-6^ M (92.5% -versus- 92.5%) respectively (Figure 8).

Both gemcitabine-(C_4_-*amide*)-[anti-HER2/*neu*] and gemcitabine chemotherapeutic each produced greater levels of anti-neoplastic cytotoxicity when they are applied in dual combination with griseofulvin (15 μM fixed-concentration) (Figure 7 and 8). Compared to gemcitabine-(C_4_-*amide*)-[anti-HER2/*neu*] alone, the chemotherapeutic gemcitabine produced greater levels of anti-neoplastic cytotoxicity when applied either alone or in dual combination with griseofulvin (15 μM fixed-concentration) (Figure 7 and 8). Greater anti-neoplastic cytotoxicity for gemcitabine in dual combination with griseofulvin compared to gemcitabine-(C_4_-*amide*)-[anti-HER2/*neu*] in dual combination with griseofulvin was evident at the gemcitabine-equivalent concentrations of 10^-10^ M (27.8% -versus- 21.1%), 10^-9^ M (45.1% -versus- 21.9%), 10^-8^ M (80.1% -versus- 23.2%), 10^-7^ M (91.2% -versus- 31.4%), and 10^-6^ M (92.5% and 55.1%) respectively (Figure 8). Gemcitabine-(C_4_-*amide*)-[anti-HER2/*neu*] in dual combination with griseofulvin produced the most rapid increases in cytotoxic anti-neoplatic potency between the gemcitabine-equivalent concentrations of 10^-8^ M and 10^-6^ M which corresponded to levels of 23.2% and 55.1% (76.8% and 44.9% residual survival) respectively (Figure 8). Gemcitabine in dual combination with griseofulvin (15 μM fixed-concentration) produced relatively rapid and progressive increases in anti-neoplastic cytotoxicity potencies from 27.8% to 91.2% (72% and 8.8% residual survival) at and between the gemcitabine-equivalent concentrations of 10^-10^ M and 10^-7^ M respectively (Figure 8). Mean maximum anti-neoplastic cytotoxicity potencies of 55.1% and 92.5% (44.9% and 7.5% residual survival) were detected for dual combinations of gemcitabine-(C_4_-*amide*)-[anti-HER2/*neu*] with griseofulvin, and gemcitabine with griseofulin respectively at the gemcitabine-equivalent concentration of 10^-6^ M (Figure 8).

## Discussion

### General

The inhibition of neoplastic cell vitality by anti-trophic immunoglobulin fractions including anti-HER2/*neu*, anti-EGFR, anti-VEGF or anti-IGF-1 is almost invariably compromised by their inability to evoke effective levels of anti-neoplastic cytotoxicity due to their tendency to promote elevated levels of cell-cycle G_1_-arrest, increased states of apoptosis-resistance [55], and selection for resistant sub-populations [1,2]. Transformations of this type can be further complicated by frequent reversal of tumor growth inhibition [1] and relapse trophic receptor over-expression [56] following discontinuation of administration. The anti-neoplastic properties of monoclonal immunoglobulin preparations that inhibit the function of trophic receptor complexes can, however, be complemented in scenarios where they are administered in combination with conventional chemotherapeutics or other cancer treatment modalities [18,19,57].

The molecular design and implementation of succinimidyl 4,4-azipentanoate in organic chemistry reactions schemes to create the UV-photoactivated gemcitabine-(C_4_-*amide*) intermediate for the synthesis of gemcitabine-(C_4_-*amide*)-[anti-HER2/*neu*] [23] or other covalent gemcitabine immunochemotherapeutics has not been extensively delineated to date. Somewhat analogous organic chemistry reaction schemes have however been described in a limited number of investigations for the synthetic production of a covalent gemcitabine-(C_5_-*methylhydroxy*)-[anti-HER2/*neu*] immunochemotherapeutic [22]. Gemcitabine-(C_4_-*amide*)-[anti-HER2/*neu*] and the organic chemistry reactions utilized in the corresponding synthesis scheme offer several distinct advantages including gentler reaction conditions, greater retained biological activity (IgG binding avidity), greater end-product yield (due to less IgG degradation or polymerization), flexibility of prolonged storage of the UV-photoactivated chemotherapeutic intermediate, and implementation of a covalent bond forming moiety that lacks any aeromatic ring structure which is known to decrease the the probability of inducting humoral immune responses.

### Anti-neoplastic cytotoxicity: Gemcitabine-[anti-HER2/neu]

Increases in the molar chemotherapeutic-equivalent concentrations of gemcitabine-(C_4_-*amide*)-[anti-HER2/*neu*] created declines in the survival of chemotherapeutic-resistant mammary adenocarcinoma (SKBr-3) populations (Figures 4,7 and 8). Cytotoxic anti-neoplatic potency of gemcitabine-(C_4_-*amide*)-[anti-HER2/*neu*] against chemotherapeutic-resistant mammary adenocarcinoma (SKBr-3) following an incubation period of 182-hours was very similar to gemcitabine alone after a shorter 72-hour incubation period (Figure 4). Gemcitabine-(C_4_-*amide*)-[anti-HER2/*neu*] at the gemcitabine-equivalent concentrations of 10^-7^ M or 10^-6^ M during a 182-hour incubation period did not exert substantially greater selectively “targeted” anti-neoplastic cytotoxicity against chemotherapeutic-resistant mammary adenocarcinoma (SKBr-3) compared to gemcitabine alone (Figures 4, 7 and 8). Such findings are in contrast to the measurably greater or equivalent levels of anti-neoplastic cytotoxicity of covalent epirubicin-[anti-HER2/*neu*] immunochemotherapeutics compared to epirubicin alone [44-47].

Conceptually there are at least five analystical variables that could have alternatively been modified to achieve substantially higher total levels of anti-neoplastic cytotoxicity for gemcitabine-(C_4_-*amide*)-[anti-HER2/*neu*]. First, incubation times with chemotherapeutic-resistant mammary adenocarcinoma (SKBr-3) could have been lengthened since longer periods of direct contact (>182-hours) appear to be indicated for covalent gemcitabine immunochemotherapeutics [22,23,28,58,59]. Longer direct contact incubation periods allow a greater opportunity for larger amounts of gemcitabine to be internalized by receptor-mediated endocytosis and subsequently liberated intracellularly from gemcitabine-(C_4_-*amide*)-[anti-HER2/*neu*] within the phagolysosome following internalization (Figure 4). Support for this consideration in based on the observation that there was a simple dose effect for gemcitabine-(C_4_-*amide*)-[anti-HER2/*neu*], and because mammary adenocarcinoma (SKBr-3) survivability was very similar when challenged with gemcibatine-(C_5_-*methylcarbamate*)-[anti-HER2/*neu*] [22] or gemcitabine-(C_4_-*amide*)-[anti-HER2/*neu*] [23] at 182-hours compared to gemcitabine at 72-hours, and increased dramatically for gemcitabine when the incubation period was extended to 182-hours (Figures 4,7 and 8) [22,23].

Second, anti-neoplastic cytotoxicity of gemcibatine-(C_4_-*amide*)-[anti-HER2/*neu*] could alternatively have been assessed against a non-chemotherapeutic-resistant human neoplastic cell type similar to those utilized to evaluate majority of the covalent biochemotherapeutics reported in the literature to date. Similarly, the cytotoxic anti-neoplatic potency of gemcibatine-(C_4_-*amide*)-[anti-HER2/*neu*] could have alternatively been measured against an entirely different neoplastic cell type that has a relatively higher sensitivity to gemcitabine such as pancreatic carcinoma [60], small-cell lung carcinoma [61], neuroblastoma [62], or leukemia/lymphoid [63,64]. In addition, human promyelocytic leukemia [28,64], T-4 lymphoblastoid clones [64], glioblastoma [28,64] cervical epitheliod carcinoma [64], colon adenocarcinoma [64], pancreatic adenocarcinoma [64], pulmonary adenocarcinoma [64], oral squamous cell carcinoma [64], and prostatic carcinoma [58] have been found to be sensitive to gemcitabine and covalent gemcitabine-(oxyether phopholipid). Within this array of neoplastic cell types both human mammary carcinoma (MCF-7/WT-2’) [64] and mammary adenocarcinoma (BG-1) [64] are known to be relatively more resistant to gemcitabine and gemcitabine-(oxyether phopholipid). Presumably this pattern of gemcitabine sensitivity is directly relevant to the cytotoxic anti-neoplatic potency detected for gemcibatine-(C_4_-*amide*)-[anti-HER2/*neu*] in chemotherapeutic-resistant mammary adenocarcinoma (SKBr-3) populations (Figure 4).

*Third*, [^3^H]-thymidine, or an ATP-based assay could have alternatively been applied to measure anti-neoplastic cytotoxicity of gemcitabine-(C_4_-*amide*)-[anti-HER2/*neu*] since they are reportedly >10-fold more sensitive in detecting early sub-lethal cell injury compared to MTT vitality stain assay methods [65,66]. Despite this consideration, MTT vitality stain continues to be extensively applied for routine assessment of true anti-neoplastic cytotoxicity in contrast to transient or sub-lethal injury for chemotherapeutics covalently incorporated synthetically into molecular platforms that provide properties of selective “targeted” delivery [28,44,64,67-73]. In this context, one distinctly important attribute of MTT vitality stain based assays is that they provide a way of measuring the extent of cell death induced by an anti-cancer agent within a population of neoplastic cells in a manner that tends to have greater relevance to clinical oncology in contrast to assays for biomarkers that simply reflect transient (non-lethal) cell injury.

*Forth*, anti-neoplastic cytotoxicity of gemcibatine-(C_4_-*amide*)-[anti-HER2/*neu*] immunochemotherapeutic could have been delineated *in-vivo* against human neoplastic xenographs in animal hosts as a model for human cancer. Many if not most covalent immunochemotherapeutics with properties of selective “targeted” delivery frequently have a higher degree of effectiveness and potency when evaluated *in-vivo* in contrast to levels acquired *ex-vivo* in tissue culture models utilizing the same cancer cell type [74-76]. Enhanced efficacy and potency is in part attributable to endogenous immune responses including antibody-dependent cell cytotoxicity (ADCC) phenomenon [77] in concert with complemented-mediated cytolysis induced by formation of antigen-immunoglobulin complexes on the exterior surface membrane of “targeted” neoplastic cell populations. During ADCC events cytotoxic components are liberated that additively and synergistically enhance the anti-neoplastic cytotoxicity activity of conventional chemotherapeutic agents [78]. Contributions of ADCC and complement-mediated cytolysis to the *in-vivo* anti-neoplastic cytotoxicity of covalent immunochemotherapeutics is further complemented by the additive and synergistic anti-neoplastic properties attained wiith anti-trophic receptor monoclonal immunoglobulin when applied in dual combination with conventional chemotherapeutic agents [18,19,79-88]. Additive or synergistic interactions of this type have been delineated between anti-HER2/*neu* when applied in dual combination with cyclophosphamide, docetaxel, doxorubicin, etoposide, methotrexate, paclitaxel, or vinblastine [19,79].

Fifth, strategies for the synthesis of gemcitabine-(C_4_-*amide*)-[anti-HER2/*neu*] could have been modified to increase the gemcitabine molar-incorporation-index. Unfortunately, such modifications usually require the implementation of harsher reaction conditions that in turn impose a higher risk of reduced biological activity (e.g. IgG antigen binding avidity) and substantial declines in final/total product yield [75,89]. Aside from overly harsh synthesis conditions, excessively high molar incorporation indexes for any chemotherapeutic agent can also reduce biological integrity of immunoglobulin fractions when the number of pharmaceutical groups introduced into the Fab’ antigen-binding region becomes excessive. Such alterations can result in only modest declines in immunoreactivity (e.g. 86% for a 73:1 ratio) but disproportionately large declines in anti-neoplastic activity in addition to substantial reductions in potency [75].

Biological integrity of the immunoglobulin component of covalent immunochemotherapeutics is critically important because it facilitates selective “targeted” delivery of the chemotherapeutic moiety and it’s subsequent internalization by mechanisms of receptor-mediated endocytosis when an appropriate “target” site on the external membrane has been selected [90,91]. Immunoglobulin-induced receptor-mediated endocytosis at membrane HER2/*neu* complexes ultimately can result in increases in the intracellular concentration of selectively “targeted”/delivered chemotherapeutic that are 8.5[91] to >100 × fold greater [92] than those attainable by simple passive diffusion. Although specific data for HER2/*neu* and EGFR expression by mammary adenocarcinoma (SKBr-3) is limited [44], other neoplastic cell types like metastatic multiple myeloma are known to internalize approximately 8×10^6^ molecules of anti-CD74 monoclonal antibody per day [93].

#### Griseofulvin anti-neoplastic cytotoxicity

The mechanism-of-action for griseofulvin is attributed to its binding-avidity for to tubulin and disruption of microtubule function resulting in an inhibition of normal mitosis [94]. In neoplastic cell types griseofulvin is known to promote suppression of centriole clustering [94], stabilization of microtubule dynamics [94], and G_2_/M arrest [95,96]. Failure of chromosomal division in turn induces tumor cell death but interestingly not in normal healthy mammalian cell populations. Griseofulvin additionally stimulates p53 activation [94] and induces apoptosis [95] that can be detected by recognizing increases in DNA fragmentation (“laddering”) [95,96] nuclear lamin alterations [95], along with induced alterations in expression profiles for Cdc2 kinase [95,96], caspase-8 [96], caspase-9 [96], and changes in viability staining characteristics [95]. Neoplastic cell types that potentially may be clinically sensitive to griseofulvin include mammary carcinoma [94,97], cervical carcinoma [97,98], colorectal carcinoma [95,96,99], oral squamous cell carcinoma [97,100], hepatocellular carcinoma [95], osteosarcoma [97], and myeloid leukemia [96].

Griseofulvin while functioning as a tubulin/microtubulin inhibitor exerted detectable levels of anti-neoplastic cytotoxicity against chemotherapeutic-resistant mammary adenocarcinoma (SKBr-3) particularly between the final concentrations of 1 μM to 20 μM and it was more potent than methylselenocysteine formulated at equivalent concentration levels (Figure 5). Increasing the incubation period from 72-hours to 182-hours measurably increased the anti-neoplastic cytotoxicity of griseofulvin (Figure 6).

#### Anti-neoplastic cytotoxicity of dual combinations

The griseofulvin mechanism-of-action is similar to the vinca alkaloids, taxanes (e.g. paclitaxel), podophyllotoxins (e.g. etoposide) and monomethyl auristatin E (MMAE). Based on these properties it can be speculated that griseofulvin has a potential capacity to additively or synergistically enhance the anti-neoplastic cytotoxicity of conventional and selectively “targeted” chemotherapeutics. Such properties have to date largely remained unknown except for limited preliminary descriptions for the dual combinations of nocodazole/griseofulvin [95,99] and vinblastine/griseofulvin [94].

Griseofulvin (15 μM fixed-concentration) consistently evoked greater levels of anti-neoplastic cytotoxicity when applied in dual combination with gemcitabine-(C_4_-*amide*)-[anti-HER2/*neu*] compared to the covalent gemcitabine immunochemotherapeutic alone at and between the gemcitabine-equivalent concentrations of 10^-10^ M and 10^-6^ M (Figure 7). The trend was most significant at 10^-10^ M and 10^-6^ M and the maximum level of anti-neoplastic cytotoxicity was 55.1% (44.9% residual survival) detected at the highest gemcitabine-equivalent concentration of 10^-6^ M (Figure 7). In an analogous manner, the anti-neoplastic cytotoxicity properties of gemcitabine was also consistently enhanced in the presence of griseofulvin (15 μM fixed-concentration) at the gemcitabine-equivalent concentrations of 10^-10^, 10^-9^ and 10^-8^ while roughly equivalent and maximum levels of anti-neoplastic cytotoxicity was observed at 10^-7^ M (91.2% -versus- 88.3%) and 10^-6^ M (92.5% -versus- 92.5%) respectively (Figure 8). Gemcitabine-(C_4_-*amide*)-[anti-HER2/*neu*] in dual combination with griseofulvin compared to gemcitabine alone exerted levels of anti-neoplastic cytotoxicity that were nearly equivalent at gemcitabine-equivalent concentrations of 10^-10^ M and 10^-9^ M and essentially equivalent at 10^-8^ M (Figure 7). Gemcitabine alone produced substantially higher levels of anti-neoplastic cytotoxicity than gemcitabine-(C_4_-*amide*)-[anti-HER2/*neu*] in dual combination with griseofulvin at gemcitabine-equivalent concentrations of 10^-7^ M and 10^-6^ M (Figure 7). The anti-neoplastic cytotoxicity for the dual combination of gemcitabine with griseofulvin was nearly equivalent to gemcitabine-(C_4_-*amide*)-[anti-HER2/*neu*] with griseofulvin at the gemcitabine-equivalent concentration of 10^-10^ M but was subtantially greater at 10^-9^ M, 10^-8^ M, 10^-7^ M and 10^-6^ M (Figure 8).

The anti-neoplastic cytotoxicity profiles for griseofulvin applied in dual combination with the covalent gemcitabine immunochemotherapeutic or gemcitabine collectively validated speculation that this alternative tublin/microtubule inhibitor can exert complementary levels of efficacy against chemotherapeutic-resistant mammary adenocarcinoma (SKBr-3) and potentially other neoplastic cell types (Figure 7 and 8). The implications of these findings are in accord with results from previous reports that recognized detectable increases in anti-neoplastic cytotoxicity activity for covalent epirubicin immunochemotherapeutics and epirubicin against chemotherapeutic-resistant mammary adenocarcinoma (SKBr-3) when applied in dual combination with griseofulvin [47]. Undoubtedly, levels of anti-neoplastic cytotoxicity for gemcitabine-(C_4_-*amide*)-[anti-HER2/*neu*] immunochemotherapeutic in dual combination with griseofulvin (15 μM fixed-concentration) would in all probability have been greater during incubation periods longer than 182-hours.

Discovery that griseofulvin (tubulin/microtubule inhibitor) can independently exert anti-neoplastic cytotoxicity activity against chemotherapeutic-resistant human adenocarcinoma, and enhance (additively or synergistically) the anti-neoplastic cytotoxicity of conventional gemcitabine and selectively “targeted” gemcitabine immunochemotherapeutics is important and has multiple implications. The combination of griseofulvin and gemcitabine-(C_4_-*amide*)-[anti-HER2/*neu*] presents a potential opportunity to attain additive and/or synergistic levels of anti-neoplastic cytotoxicity through three different molecular mechanisms (e.g. griseofulvin/gemcitabine, griseofulvin/[anti-HER2/*neu*], and gemcitabine/[anti-HER2/*neu*] dual combination effects). Attributes of this nature are at least in part complemented by both griseofulvin [101] and the chemotherapeutic moiety of covalent immunochemotherapeutics [27,28] like gemcitabine-to-[anti-HER2/*neu*] functioning as poor P-glycoprotein substrates. Griseofulvin in dual (additive or synergistic) combination with either a covalent gemcitabine-(C_4_-*amide*)-[anti-HER2/*neu*] or gemcitabine therefore offer the option for developing treatment schemes that potentially evoke more rapid and long-term (durable) resolution of even chemotherapeutic-resistant neoplastic cell populations. Complementary qualities that griseofulin in dual (additive or synergistic) combination with gemcitabine-(C_4_-*amide*)-[anti-HER2/*neu*] or gemcitabine ultimately can afford are; [i] lower total chemotherapeutic dosage requirements; [ii] reduced frequency and severity of sequelae, and a [iii] decreased probabilty of complete therapeutic resistance. Fewer and less severe sequelae are at least conceptually probable because of the relatively wider marginof-safety of griseofulvin compared to many if not most conventional chemotherapeutics [102-105] which is further complemented by the selective “targeted” delivery properties of gemcitabine-(C_4_-*amide*)-[anti-HER2/*neu*] and related covalent immunochemotherapeutics. Lastly, application of griseofulvin as an alternative tubulin/microtubule inhibitor in dual combination with either a covalent gemcitabine immunochemotherapeutic or gemcitabine is in direct accord with the general recommendation for *in-vivo* treatment regimens. Current clinical oncology guidelines advocate that different anti-cancer agents administered during the course of multi-chemotherapeutic schedules ideally should exert distinctly different mechanisms-of-action (avoids competitive inhibition) and individually evoke different sets of undesirable sequellae.

## Conclusion

Organic chemistry reaction schemes are now available that can facilitate the synthesis of gemcitabine-(C_4_-*amide*)-[anti-HER2/*neu*] and related covalent gemcitabine immunochemotherapeutics. Qualities of the synthesis method include; [i] greater flexibility for conveniently bonding gemcitabine other chemotherapeutics to selective “targeted” delivery platforms at a greater chemotherapeutic molar incorporation index; [ii] posses less risk of spontaneous immunoglobulin polymerization compared to methods that require pre-thiolation; [iii] posses less risk of promoting low-molecular-weight fragmentation; and can be [iv] utilized as a model for the design and synthesis of covalent chemotherapeutic-ligands or immunochemotherapeutics that employ different selective “targeted” delivery platforms and other chemotherapeutic agents.

Anti-neoplastic cytotoxicity potencies for gemcitabine-(C_4_-*amide*)-[anti-HER2/*neu*] at the end of a 182-hour incubation period were similar to gemcitabine following a 72-hour incubation period in populations of chemotherapeutic-resistant mammary adenocarcinoma (SKBr-3). Anti-neoplastic cytotoxicity of gemcitabine-(C_4_-*amide*)-[anti-HER2/*neu*] would likely have been greater if it had been evaluated using an incubation period greater than 182-hours or had been determined against human promyelocytic leukemia, T-4 lymphoblastoid clones, glioblastoma; cervical epitheliod carcinoma, colon adenocarcinoma, pancreatic adenocarcinoma, pulmonary adenocarcinoma, oral squamous cell carcinoma, or prostatic carcinoma.

The molecular design of gemcitabine-(C_4_-*amide*)-[anti-HER2/*neu*] and related covalent gemcitabine immunochemotherapeutics [22,23] result in the synthesis of an anti-cancer preparation that affords a more prolonged plasma pharmacokinetic profiles for the gemcitabine moiety. In the form of a covalent immunochemotherapeutic, gemcitabine has a substantially longer plasma half-life (T_1/2_) that is at least in part attributable to; [i] a reduced gemcitabine deamination within gemcibatine-(C_4_-*amide*)-[anti-HER2/*neu*] due to substrate steric hinderance phenomenon; and [ii] decreased gemcitabine renal clearance rate [24-26] due to the substantially larger molecular weight for gemcibatine-(C_4_-*amide*)-[anti-HER2/*neu*] (MW ~ 150 kDa) compared to gemcitabine (MW=263.2) which far exceeds the molecular weight cutoff for excretion by glomerular filtration. Prolongation of the pharmacokinetic profiles for gemcitabine in the form of gemcitabine-(C_4_-*amide*)-[anti-HER2/*neu*] ultimately complements, enhances and facilitates the properties of selective “targeted” chemotherapeutic delivery, continual cancer cell membrane deposition, and progressive intracellular chemotherapeutic accumulation.

Anti-neoplastic cytotoxicity of gemcitabine or gemcitabine-(C_4_-*amide*)-[anti-HER2/*neu*] was increased when they were applied in dual combination with the tubulin/microtubule inhibitor griseofulvin against chemotherapeutic-resistant mammary adenocarcinoma (SKBr-3) populations. The implications of this discovery are important and wide-ranging in scope because they they offer the potential option for developing treatment schemes that more rapidly evoke durable (longterm) resolution of neoplastic disease states while simultaneously providing certain properties that impose a lower frequency and severity of sequelae and susceptibility to resistance. More rapid resolution at least in theory could be achieved with gemcitabine-(C_4_-*amide*)-[anti-HER2/*neu*] or gemcitabine in dual combination with with the tubulin/microtubule inhibitor griseofulvin because of the additive or synergistic levels of anti-neoplastic cytotoxicity activity that could be attained that in turn will also lower total dosage requirements and total dose administered. Lower frequency of resistance is attained with gemcitabine-(C_4_-*amide*)-[anti-HER2/*neu*] and other chemotherapeutic analogs that are covalently bound to large molecular weight platforms are apparently poor substrates for P-glycoprotein/MDR-1 (multi-drug resistance efflux pump) [27,28]. Similar in concept to the benzimidazole tubulin/microtubule inhibitors [106-108], griseofulvin may potentially be a poor P-glycoprotein/MDR-1 substrate but this property remains to be more concisely delineated. Given this perspective, resistant forms of breast cancer that over-expresses EGFR and HER2/*neu* are often less vulnerable to the cytotoxic potency of chemotherapeutics due to a simultaneous over-expression of trans-membrane P-glycoprotein which functions as a somewhat non-selective membrane “pump” complex for many pharmaceutical agents [109-114].

Greater levels of safety could potentially be attained through several different avenues. Covalent gemcitabine immunochemotherapeutic or gemcitabine in dual combination with the tubulin/microtubule inhibitor, griseofulvin directly coincides with the general recommendation for *in-vivo* treatment regimens from a clinical oncology perspective. Such guidelines in part advocate that different anti-cancer agent classes administration of during the course of multi-chemotherapeutic regimens should ideally exert different mechanisms-of-action (avoids competitive inhibition) and individually precipitate distinctly different sets of undesirable sequellae. In such dual combinations, enhanced levels of safety are naturally attained with gemcitabine-(C_4_-*amide*)-[anti-HER2/*neu*] because of their properties of selective “targeted” delivery that avoid innocent tissue/organ system exposure and lower total dosage requires associated with attaining additive or synergistic levels of anti-neoplastic cytotoxicity efficacy. Griseofulvin would complement the lower frequency and severity of sequelate associated with gemcitabine-(C_4_-*amide*)-[anti-HER2/*neu*] because this tubulin/microtubule inhibitor is has a wider margin of safety than many if not most conventional chemotherapeutics or other pharmaceutical agents [115-117]. Conventional tubulin/ microtubule inhibitor chemotherapeutics include colchicine [118], the vinca alkaloids [119-123], taxanes (e.g. paclitaxel) [123-125], podophyllotoxins (e.g. etoposide: semi-synthetic derivative) [126,127] and monomethyl auristatin E (MMAE) [128]. The narrow margin of safety (therapeutic index) and relative lack of efficacy for colchicine restricts its wide-spread application for breast cancer treatment [118,129,130]. Administration of the vinca alkaloid tubulin inhibitor chemotherapeutics (e.g. vinblastine, vincristine) is often complicated by neurotoxicity [131-134], bone marrow suppression [135,136], and emergence of therapeutic resistance patterns [123,133,137-143]. The taxane class of anti-tubulin chemotherapeutics (e.g. paclitaxel, docetaxael) suffers from very similar disadvantages that includes acquired and intrinsic tumor resistance [120,123,140,141] secondary to the over-expression of multidrug resistance proteins (e.g. P-glycoprotein) [110,144,145], hypersensitivity reactions [146-149], hematopoietic toxicity (dose limiting feature) [150-152], and cumulative neurotoxicity [133,134,153] which can all curtail administration of treatment protocols [154]. Podophyllotoxins (e.g. etoposide semi-synthetic derivative) induce a moderately high frequency of dose-limiting myelosuppression (leucopenia) [126,155,156] and gastrointestinal disturbances (nausea, vomiting, stomatitis). Monomethyl auristatin E (MMAE) is far too toxic for direct systemic administration so instead it must be covalently bound to immunoglobulin (e.g. anti-GPNMB/anti-CRO11/glembatumumab and anti-CD30/brentuximab) or other similar large molecular weight “carrier” platform.

## Abbreviation

HER2/*neu*: Trophic Membrane Receptor Complex Over-expressed by Several Neoplastic Cell Types
SKBr-3: Chemotherapeutic-resistant Mammary Adenocarcinoma
MTT (vitality stain): 3-[4,5-Dimethylthiazol-2-yl]-2,5-diphenyl Tetrazolium Bromide
EDTA: Ethylene Diamine Tetra Acetic Acid
Cell-ELISA: Enzyme Linked Immunodetection Assay for Antigen (anti-HER2/*neu*) Bound to HER2/*neu* on the Exterior Surface Membrane of SKBr-3 Populations
